# Psychophysiological and Psychosocial Profile of Patients Attending Drug Addiction Centers

**DOI:** 10.1007/s10484-021-09531-1

**Published:** 2021-12-27

**Authors:** Vicente Javier Clemente-Suárez, Pablo Ruisoto, Manuel Isorna-Folgar, Jesús Cancelo-Martínez, Ana Isabel Beltrán-Velasco, José Francisco Tornero-Aguilera

**Affiliations:** 1 Faculty of Sports Sciences, Universidad Europea de Madrid, Tajo Street, s/n, Villaviciosa de Odón, 28670 Madrid, Spain; 2 Studies Centre in Applied Combat (CESCA), Toledo, Spain; 3 Grupo de Investigación en Cultura, Educación y Sociedad, Universidad de la Costa, Barranquilla, Colombia; 4 Department of Health Sciences, Public University of Navarre, Pamplona, Spain; 5 Facultad Ciencias Educación y Trabajo Social, Universidad de Vigo, Ourense, Spain; 6 Unidad Asistencial Alborada, Vigo, Pontevedra Spain; 7 Psychology Department, Universidad Antonio de Nebrija, Madrid, Spain

**Keywords:** Drug addiction, Heart rate variability, Stress, Psychosocial, Psychological trait

## Abstract

Drug treatment centres provide the highest level of rehab services for patients diagnosed with drug addictions. Most inpatient drug rehab programs focus on medical detox and mental health interventions. However, how to optimize the later remains a challenge. The aim of this study was to examine the psychophysiological and psychosocial profile of patients attending drug addiction centres in comparation with the general population. A total of 105 inpatient drug rehab patients and 50 participants from the general population were compared based on standardized psychophysiological and psychosocial measures. Results of this study suggest that patients attending drug addiction centers differ from general population in several different psychophysiological and psychosocial factors. Patients reported significantly lower levels of physical activity and increased sympathetic responsiveness, and significantly higher levels in loneliness, psychologically inflexibility and neuroticism. The results of this study highlight the importance of address healthy lifestyle behaviors such as sport practice and psychological variables such as loneliness, psychological (in)flexibility and neuroticism to improve current programs aim to prevent or reduce problematic drug consumptions.

## Introduction

Drug abuse is a disruptive behavior, being the principal approximation to this public health problem the as a disease or a learned, maladaptive behavioral pattern (Kumar et al., 2013). When it chronifies becoming an addiction, leads to a dependence syndrome, a cluster of behavioral, cognitive, and physiological phenomena which involves a strong desire to take the drug, and difficulties controlling its use. Its psychophysiological costs are a linear significant decrease of both physical and mental health. Psychosocially, it increases family income, violence, security problems, traffic, and workplace accidents, while increasing health care system cost (Kumar et al., 2013). Since it is estimated that 5.2 percent of the global population between the ages of 15 and 64 years (over 360 million people) used an illicit drug in 2014 (24 million more since 1990) drug dependence needs to be recognized in developing countries as a significant public health problem and literature on the magnitude of this problem is yet limited (Statista., 2021).

Drugs consumption produces a psychophysiological effect similar than other physiological stressor producing among them dysregulation of the autonomous nervous system (Bustamante-Sánchez et al., 2020), increasing the sympathetic nervous system branch, and decreasing the parasympathetic nervous system modulation, (Lin et al., 2016). Yet, if drug use is chronified, sympathetic activity will remain over-activated, given the addictive loop of drug use and its effect (Elghozi et al., 2001) This situation could present different effect in some cortical regions such as those related to self-perception and decision-making Clemente-Suárez et al., 2017; Delgado-Moreno et al., 2017; Bustamante-Sánchez et al., 2020. In addition, the chronic sympathetic over-activation allows to the perpetuation of antisocial behaviour, self-destructive and addictive behaviours, reinforcing the consumption of drugs and leading to a vicious circle (Stewart & Conrod, 2007).

In this line, physical exercise can modulate the autonomic nervous system response in the short, medium, and long term (Fischer et al., 2012). Thus, the implementation of regulated physical activities may be a key factor for drug consumers, especially considering their low levels of physical activity (Clemente-Suarez & Ruisoto-Palomera, 2020). It was shown how physical activity improves performance of neurotransmitters in the brain and reduces feelings of loneliness, anxiety, and depression, generating both physical and perceived well-being (Clemente-Suarez & Ruisoto-Palomera, 2020). Furthermore, recent epidemiological studies consistently report that aerobic exercise and higher levels of physical activity are inversely related to substance use and abuse (Smith & Lynch, 2011). Therefore, physical activity is presented as a non-pharmacological intervention to improve autonomic dysregulation of drug consumers, fact that could improve their antisocial behaviours. However, its implications in the prevention and treatment of drug abuse are yet unknown.

The psychological profile, personality traits and personal patterns have been also related with addiction behaviours (Kotov et al., 2010; Fehrman et al., 2019). In this line, neuroticism is the most health-related personality trait associated with a higher risk of substance abuse and other mental diseases (Jeronimus et al., 2016); as well as impulsivity (Zilberman et al., 2018). By contrary, conscientiousness was the strongest personality predictor of reduced mortality (Stephan et al., 2019) and protective health behaviours (Shanahan et al., 2014). Furthermore, loneliness, psychological inflexibility, and stress-reactivity, are also critical factors related to mental health and substance abuse (Fehrman et al., 2019). Other traits, such as openness to experience, agreeableness, and extraversion are less consistent in the literature, but appear to influence addictions and substance types (Kotov et al., 2010; Stephan et al., 2019; Shanahan et al., 2014).

The explanation for the association between substance abuse and personality traits, its interactions with the level of physical activity and autonomic modulation presents some difficulties since possible correlations have yet not been studied. Also, most of the scientific literature have been focused on specific substances or behavioural addictions, or each factor as an independent variable, limiting the potential explanations and the comprehension of a multifactorial complex health problem. Therefore, the present study aimed to analyse the psychophysiological and psychosocial profile of patients attending drug addiction centres. Our hypothesis was that patients attending drug addiction centres would present higher autonomous sympathetic modulation and a different psychological and psychosocial profile than a non-attending drug addiction centres control group.

## Material and Methods

### Participants

A total of 105 participants (71 males and 34 females) under drug addiction treatment in an addiction treatment center and a non-clinical control group of 50 participants (27 males and 23 females) were analyzed using a number of sociodemographic, psychophysiological and psychosocial measures. The study was conducted according to the principles expressed in the Declaration of Helsinki and approved by the direction of the addiction treatment center. All the participants signed a written informed consent prior to start the study and they were free to leave the study at any moment.

### Design and Procedure

A descriptive cross-sectional study was conducted. We analyzed the autonomic modulation of participants by the Heart Rate (HR) and Heart Rate Variability (HRV). For this aim, participants were in a quiet and temperature-controlled room (22.1 ± 0.5 ºC, 40% of humidity), sited in a chair in a comfortable position and without speaking. The participants were 15 min in this position, but only the 10 last minutes were used for analysis. The HR and HRV was recorded with the Polar V800 HR monitor (Kempele, Finland). with a sampling frequency of 1000 Hz, that allow to monitor the RR intervals (time interval between R waves of the electrocardiogram) for the analysis of the HRV and the number of beats per minute for the HR analysis. Subsequently, HRV parameters analyzed using the Kubios HRV software program v.3.0 (University of Kuopio, Kuopio, Finland). Three HRV domains were included as indicators of autonomic stress reactivity:

Time-Domain (Nonspectral) Analysis. This analysis was based on the assessment of the intervals between normal beats on ECG recordings. During the statistical analysis, generally all the QRS complexes, the duration between consecutive QRS complexes (NN interval), or the instantaneous heart rates during continuous ECG recordings are determined. We recorded the following time-domain indices: pNN50 which is the percentage of successive normal sinus RR intervals exceeding 50 ms (%) and RMSSD (ms): Is the square root of the mean value of the sum of squared differences of all successive R-R intervals.

Frequency-Domain (Spectral Measures) Analysis. Frequency-domain measures give information about how the power is distributed as a function of frequency. This analysis give us smoother spectral components that can be distinguished as independent from preselected frequency bands and easy post-processing of the spectrum with an automatic calculation of low (LF)- and high-frequency (HF) power components and an easy identification of the central frequency of each component, and accurate estimation even on a small number of samples (Bustamante et al., 2020).HF and LF (n.u) were measured in order to measure the peaks of parasympathetic, high-frequency component, frequency range: 0.15–0.40 Hz (HF) and sympathetic low-frequency component frequency range: 0.04–0.15 Hz (LF) values. In addition, LF/HF ratio was evaluated.

Nonlinear domain. SD1 and SD2 were measured to reflect the fluctuations of the HRV throw a Poincaré chart, physiologically, the transverse axis. SD1 reflects parasympathetic activity while SD2 reflect the long-term changes of RR intervals and is considered as an inverse indicator of sympathetic activity. In addition, the Approximate Entropy (ApEn) and Sample Entropy (SampEn) of the Heart Rate Variability were measured.

After the HR and HRV analysis participants went to a different room to fill a battery of psychological questionnaires with the presence of a professional to solve any questions they may have. The questionnaires were as follows.

*Avoidance and Action Questionnaire (AAQ-7)* (Bond et al., 2011). This is the most widely used general measure of psychological inflexibility, defined as rigidity in the handling of emotions or unpleasant internal events. It consists of 7 items and participants respond to a 7-point Likert-type scale, from 1 = “never” to 7 = “always”. Scores range from 7 to 49. Higher scores indicate tendency to act under the need to control or avoid aversive thoughts, memories, or feelings. Cronbach’s alpha coefficient for internal consistency reliability was α = 0.93 for males and α = 0.95 females.

*UCLA Loneliness Scale Revised-Short* (Hughes et al., 2004). This consists of a brief 3-item scale evaluating the subjective feeling of loneliness, understood as the perception of less social support being available than desired. Participants respond based on their agreement with previous statements, 1 = “hardly ever”, 2 = “sometimes”, and 3 = “often”. Scores range from 0 to 9. Higher scores indicate greater feeling of loneliness or lack of social support. Cronbach’s alpha coefficient for internal consistency reliability was α = 0.76 for males and α = 0.84 for females.

*Big Five Inventory-10* (Rammstedt & Jhon, 2007). It consists of a 10-item abbreviated version of the original 44-item Big Five Inventory. It assesses extraversion, agreeableness, conscientiousness, neuroticism, and openness to experience. Participants respond on a 5-points Likert type scale ranging from, 1 = “strongly disagree”, to 5 = “strongly agree” based on self-reports of how well a number of 10 statements describe their personality. Scores range from 10 to 50. Cronbach’s alpha coefficient for internal consistency reliability was α = 0.75.

Sociodemographic data. We asked for the participants age (years), body mass index (Kg/m^2^), weekly physical training (h), movement per day (min), sleeping time (h) and the relationship with their parents (0 to 10 scale).

### Statistical Analysis

All data analyses were performed using the Statistical Package for the Social Sciences, version 21 for Mac (IBM Spain, Madrid, Spain). The descriptive analysis of the sample included the means and standard deviations (M + SD) for the quantitative variables, while frequencies and percentages were used for the nominal variables. Normality and homoscedasticity of variance were examined using Shapiro–Wilk and Levene´s test respectively. Students´ t tests were conducted to explore differences in psychosocial (personality traits, psychological inflexibility, and loneliness) and physiological variables (stress reactivity) between patient attending addiction centers vs control group.

## Results

### Sociodemographic Description of the Sample

The group of patients attending addiction centers reported their first consumption at the age of Md = 16 years old (IQR = 4.75) and have been involved in the program for the last 8.33 years under rehabilitation program (IQR = 1.19). A total of 67,47% were unemployed. 44,76% had basic education, 53.3% secondary education. A total of 86.67 were smokers and 71% were policonsumers. The first drug of abuse was the following: cocaine (27.62%), heroin (20%), cannabis (9.52%), alcohol (8,57%), hashish (4,76%), tobacco (1.90%), opioids (0,95%) and amphetamines (0,95%). A total of 38.09% reported doing exercise regularly and 78.09% having a healthy diet. The control group was characterized by workers (100%) with university academic training (100%) and no history of drug consumption, although a 6% reported that smoked cigarettes. A total of 62.00% reported doing exercise regularly and 96% having a healthy diet.

### Sociodemographic Differences of the Sample

The results are reported as mean ± SD. Only differences between control and patients’ group were seen regarding physical activity behaviors. Control group presented greater significant hours of weekly physical training and minutes of movement per day than patients’ group (Table 1).

### Psychosocial and Physiological Differences of the Sample

The group of patients attending addiction centers reported all greater significant values of Extraversion (BFI- E); Agreeableness (BFI-A); Neuroticism (BFI-N); Psychological inflexibility (AAQ II) and Loneliness (UCLA), than control group. Factors that make drug use prone, characterizing the psychological profile of patients (Table 2).

According to the HRV analysis, patient group present significant lower values of ApEN, SampEn and Pnn50, while higher HRmin than control group (Table 3).

**Fig. 1:**
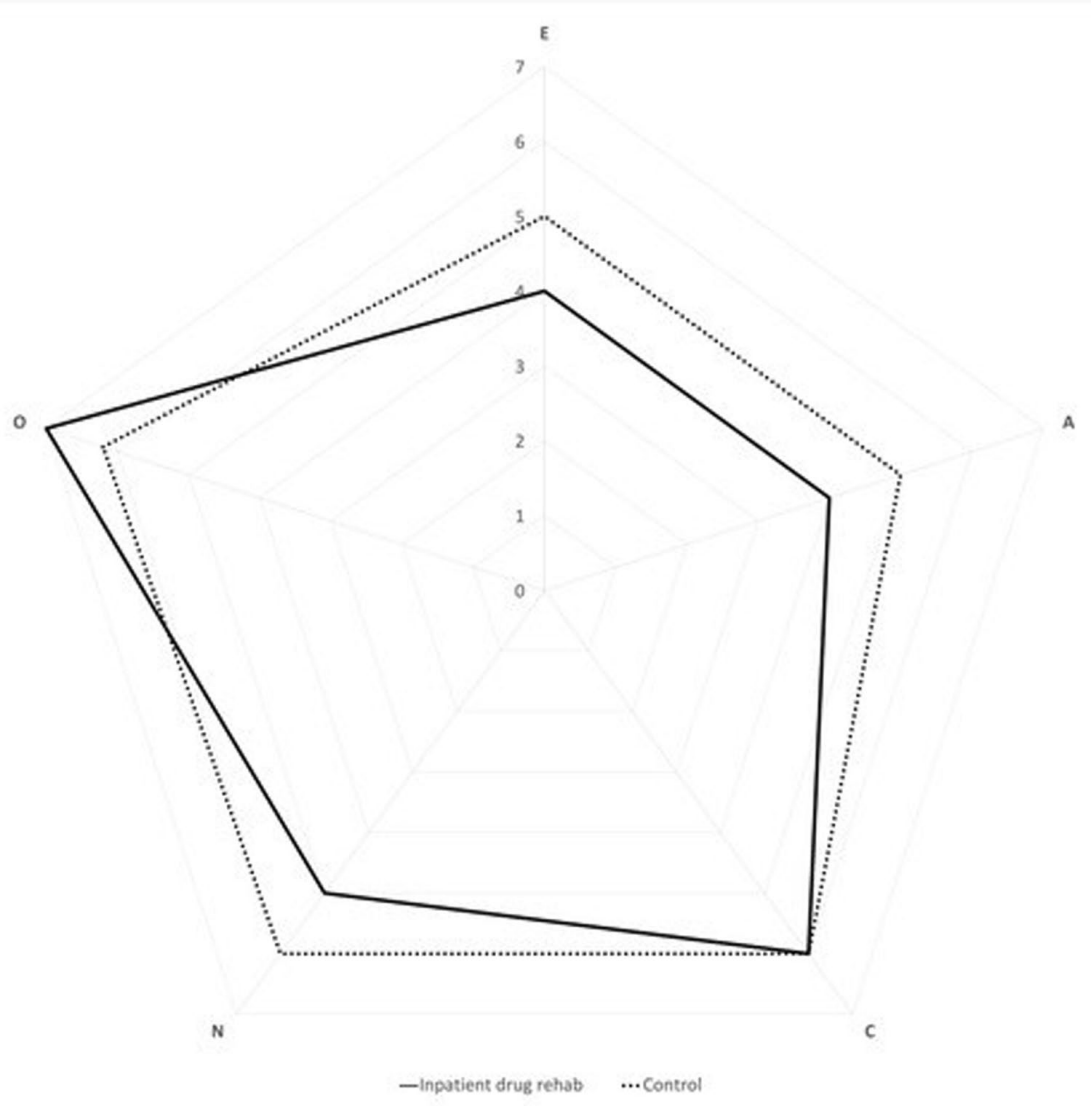
Radar diagram for differences in personality profile between people in addiction centers and controls. *A* agreeableness, *E* extraversion, *N* neuroticism, *C* conscientiousness, *O* openness to experience

The diagram of the 5 BFI personality traits suggest patient group has a higher score of extraversion, agreeableness, and neuroticism while similar openness to experience and conscientiousness than patients’ group, which is in accordance with the results of the Table 2 (Fig. 1).

**Table 1:** Differences between patients and control group in sociodemographic variables

	*Patients´ Group* (n = 105)		*Control Group* (n = 50)		*p*	*Cohen´s d*
	*M*	*SD*	*M*	*SD*		
Age (years)	39.84	6.43	41.94	9.52	0.160 n.s	− 0.243
Body mass index (kg/m^2^)	23.46	2.31	24.67	4.55	0.077 n.s	− 0.306
Weekly physical training (hours)	2.97	1.36	3.27	2.00	< .001**	0.616
Movement per day (min)	105.00	2.163	360.00	− .056	< .001**	− 1.384
Sleep (h)	7.032	0.67	7.332	1.87	0.262 n.s	− 0.195
Relationship with parents (0–10)	7.77	1.69	7.62	2.40	0.695 n.s	0.068

n.s. = no significant; *p < .05; **p < .001

**Table 2:** Differences between patients and control group in psychosocial variables

	*Patients´ Group* (n = 105)		*Control Group* (n = 50)		*p*	*Cohen´s d*
	*M*	*SD*	*M*	*SD*		
Extraversion (BFI-E)	5.55	1.80	4.12	1.69	< .001 **	− 0.814
Agreeableness (BFI-A)	5.48	2.11	4.02	1.74	< .001**	− 0.732
Conscientiousness (BFI-C)	5.93	2.18	6.22	1.79	0.422 n.s	0.138
Neuroticism (BFI-N)	5.82	2.55	4.96	2.23	0.041 **	− 0.353
Openness to experience (BFI-O)	6.17	2.55	4.96	2.23	0.256 n.s	0.196
Psychological inflexibility (AAQ II)	26.87	10.11	16.04	6.27	< .001 **	− 1.196
Loneliness (UCLA)	4.77	2.03	3.64	0.96	< .001 **	− 0.640

n.s. = no significant; *p < .05; **p < .001

**Table 3:** Differences between patients and control group in physiological variables (stress-reactivity)

HRV dimensions	*Patients´ Group* (n = 105)		*Control Group* (n = 50)		*p*	Cohen´s *d*
	M	SD	M	SD		
*Temporal*						
HR min	68.89	14.78	57.91	15.81	< .001**	− 0.726
HR max	91.178	19.89	90.08	16.36	0.737	− 0.058
HR med	77.85	14.25	73.26	12.60	0.054	− 0.334
RMSSD	74.60	19.27	87.12	67.01	0.656	0.077
PNN50	12.48	18.26	20.37	14.98	0.009	0.457
*Frequency*						
LF/HF (ms)	4.08	6.74	3.05	2.43	0.308	− 0.178
LF (ms)	1943.30	5416.54	1509.65	1326.91	0.586	− 0.095
HF (ms)	2349.24	9877.44	711.85	903.12	0.254	− 0.200
LF (un)	66.49	19.31	70.12	12.91	0.231	0.207
HF (un)	33.30	19.20	33.30	16.17	1.000	−
VLF (ms)	221.132	789.31	167.22	153.28	0.644	− 0.081
*Non-lineal*						
SD1	53.22	136.73	61.713	47.39	0.670	0.073
SD2	78.97	209.37	88.91	52.82	0.742	0.057
ApEn	1.065	.241	1.736	2.296	0.004	0.507
SampEn	1.393	.454	1.948	2.603	0.037	0.363

n.s. = no significant; *p < .05; **p < .001

#### Discussion

This study provides a complete psychophysiological and psychosocial profile of patients undergoing treatments in an addiction center. To our knowledge, this is one of the first studies to approach from a holistic perspective the complete profile of the patients. The initial hypothesis was compiled since differences were found in the autonomous sympathetic modulation, and psychological and psychosocial profile between drug addiction patients and control group.

Patients attending drug addiction centers presented a significantly less physical activity levels and formal education background than controls, and significantly higher scores in extraversion, agreeableness, and neuroticism. Higher scores in neuroticism are particularly important, because suggest a tendency for stress reactivity, in line with previous literature (Lahey, 2009). Patients also reported higher degree of both psychological inflexibility and loneliness. This result is consistent with previous studies which suggest the importance of psychological flexibility and social support to better cope with daily emotional stressors (Luoma et al., 2011).

Physical activity levels were higher in control group, indicated by the significant higher values of weekly physical training hours and minutes of movement. This result is consequent with previous studies comparing physical activity levels of inmate’s acute drug users, and chronic drug users (Fischer et al., 2012). In addition, this result is consequent with the epidemiological studies which report that aerobic exercise and greater levels of physical activity are inversely related to substance use and abuse (Smith & Lynch, 2011). In this line, there is enough preclinical evidence showing that physical activity serves as both a preventive and treatment intervention, reducing drug use. Explanation behind this may be addressed since various brain systems are altered by physical activity, with the medial prefrontal cortex serving as one potential node that may mediate the putative link between physical activity and drug abuse vulnerability (Bardo & Compton, 2013). Interestingly, recent studies suggested that patients, especially in young ages, are actively involved in taking care of their health, which seems an important corner stone and opportunity to implement physical activity strategies and interventions as a tool for drug prevention and treatment (Drumm et al., 2005). Therefore, novel neurobehavioral approaches considering quantification, evaluation and promotion of physical activity are essential in further interventions. Primary care providers, walk-in clinics, drug treatment programs, outreach workers and those engaged in harm reduction efforts shall be highly benefited from it.

Regarding the autonomous nervous modulation, the HRV of drug users is impaired and downregulated in in comparison to healthy controls, in line with previous literature (Quintana et al., 2013). This physiological trait produces a continuous state of alarm because of the sympathetic hyperarousal. This fact is reflected in the lower PNN50, ApEn and SampEn values. These values provide quantitative information about the complexity of or reduction in the chaotic behavior of the signal of both short-term and long-term data recordings. In line with the study of Krstacic et al. (2007), lower ApEn indexes are attributed to the signal's loss of complexity and irregularity due to reduced HRV, increased sympathetic modulation and decreased vagal modulation. Furthermore, present control group data are consequent with the results of Acharya et al. (2004) who observed ApEn values of 1.68 for healthy, middle-aged individuals, as our control group, 1.73. Yet, high values of SampEn indicate high irregularity and complexity, which according to authors is associated with less internalizing psychopathology, less aggression and frustration (Fiskum et al., 2018). Furthermore, authors suggest a positive relationship between higher SampEn and better effortful control, a protective factor against psychopathology linked to frontal function (Fiskum et al., 2018). This fact is consequent with the lower significant values of patient group. Finally, according to temporal domain pNN50 value, lower values are also associated with greater sympathetic activation, and greater stress reactivity and anxiety (Liu et al., 2020). This is also in line with the significant lower pNN50 of patients.

Interestingly, control group presented a lower and significant resting heart rate (HRmin), which is in line with the significantly higher levels of physical activity, either daily minutes and hours of weekly training, denoting a higher cardiovascular capacity (Solar & Irwin, 2010). Likewise, it could be explained due to possible dilated cardiomyopathy, altered chamber dimensions and systolic function, due to chronic consumption of stupefying substances, which lead to increased cardiovascular output, thus altered maximum and minimal heart rate (Billman et al., 2015).

Results describing the profile of patients in drug addiction centers are important for two main reasons. First, allow to target interventions aim to prevent drug addiction based on these psychosocial variables prevalent in this population. Second, provide insights that enhance the design and implementation of interventions in this population. For example, individuals with higher scores in neuroticism, psychological flexibility and loneliness should be considered at risk of developing drug abuse related problems. Moreover, based on results of this study, interventions and treatments should incorporate psychosocial variables such as learn how to cope with stressful or aversive emotional and private events (psychological flexibility), increase perceived social support and formal education of patients. Overall, these results are consistent with the importance of moving beyond classical health care treatment towards interventions based on social determinants of health (Meyer et al., 2018). Yet, there are some limitations due to the sociodemographic differences between the experimental and control group, despite subjects were the same age, they had different background (university training). Finally, we can propose as a practical application the implementation of physical activities in this population in order to improve their autonomic dysregulation, specially that related with high intensity interval training structured in a reverse periodization, that allow to a decrease in sympathetic modulation (Clemente-Suárez et al., 2015; Clemente-Suárez, & Arroyo-Toledo, 2017, 2018).

Therefore, we can conclude that patients attending drug addiction centers differ from general population in several different psychophysiological and psychosocial factors. On one hand, patients reported significantly lower levels of physical activity and increased sympathetic modulation. On the other hand, patients reported significantly higher levels of loneliness, psychologically inflexibility and neuroticism, associated with increased risk of both drug-related problems and mental health problems in general. Yet, the results of this study highlight the importance of address healthy lifestyle behaviors such as sport practice and psychological variables such as loneliness, psychological (in)flexibility and neuroticism to improve current programs aim to prevent or reduce problematic drug consumptions.

## Data Availability

The data that support the findings of this study are available from the corresponding author upon reasonable request.
